# Onasemnogene Abeparvovec in Patients With SMA: Interim Results of the RESTORE Registry in Japan

**DOI:** 10.1002/acn3.70437

**Published:** 2026-06-09

**Authors:** Kayoko Saito, Kamal Benguerba, Ken Tsuchida, Kazushige Yazawa, Isao Tsumiyama, Hiromitsu Kayama, Sandra P. Reyna, Farid Khan, Richard S. Finkel

**Affiliations:** ^1^ Tokyo Women's Medical University Tokyo Japan; ^2^ Novartis Pharma AG Basel Switzerland; ^3^ Clinical Development Novartis Pharma K.K. Tokyo Japan; ^4^ Analytics Novartis Pharma K.K. Tokyo Japan; ^5^ Medical Affairs Novartis Pharma K.K. Tokyo Japan; ^6^ Novartis Gene Therapies Inc. Bannockburn Illinois USA; ^7^ Center for Experimental Neurotherapeutics St. Jude Children's Research Hospital Memphis Tennessee USA

**Keywords:** Japanese, onasemnogene abeparvovec, post‐marketing surveillance, RESTORE registry, spinal muscular atrophy

## Abstract

**Objective:**

There are limited real‐world data regarding the safety and effectiveness of onasemnogene abeparvovec (OA; Zolgensma) infusion, a one‐time gene replacement therapy, for Japanese patients with spinal muscular atrophy (SMA). We aimed to improve understanding of the real‐world outcomes for OA in Japan.

**Methods:**

We report interim, 5‐year results of Japanese post‐marketing surveillance of OA (part of the RESTORE registry: NCT04174157).

**Results:**

Eighty patients were registered and treated with OA (monotherapy: 30%; bridge or switch to OA: 54%). The median (min, max) age (months) was 3.0 (0, 18) at symptom onset and 10.0 (0, 24) at OA infusion. Forty patients each (50.0%) had two or three survival motor neuron 2 (*SMN2*) gene copies. Ten patients were identified by newborn screening. Adverse events related to OA were reported in 98.8% (serious: 26.3%; no deaths). Adverse events of special interest occurred in 92.5%, including hepatotoxicity (90.0%), transient thrombocytopenia (62.5%), cardiac adverse events (33.8%), and thrombotic microangiopathy (5.0%). Event‐free survival at 3 years since OA administration was 93.0%. There was one death from disease progression. Of 39 patients with two or more developmental milestones, 64.1% achieved new developmental milestones and 15.4% maintained their milestones. Children's Hospital of Philadelphia Infant Test of Neuromuscular Disorders scores increased by ≥ 4 points in 81.8% (54/66).

**Interpretation:**

The safety profile of OA in Japanese patients with SMA mirrored that of earlier studies. In our real‐world observations, patients showed gains in or maintenance of motor milestones or motor function scores that were sustained over the observation period.

**Trial Registration:**

NCT04174157 (ClinicalTrials.gov)

## 1 Introduction

Spinal muscular atrophy (SMA) is a severe disease caused by deletions or mutations in the survival motor neuron 1 (*SMN1*) gene [1], leading to impaired production of SMN protein.

In Japan, the reported prevalence of SMA is 1.17 cases per 100,000 people and its incidence is 0.51 in 10,000 live births. Most cases are diagnosed before 2 years of age, and clinical diagnosis is usually accompanied by a genetic diagnosis [2, 3]. Some cases are diagnosed during pregnancy or through newborn screening (NBS) [4, 5].

Onasemnogene abeparvovec (OA; Zolgensma, Novartis) is a one‐time gene replacement therapy approved in Japan and other countries for the treatment of SMA. It delivers human *SMN1* cDNA to achieve sustained expression of functional SMN protein in motor neurons, including the central nervous system and peripheral organs [6]. Multiple clinical trials [7, 8, 9, 10, 11, 12] and real‐world studies [13, 14, 15, 16, 17, 18, 19, 20] have demonstrated its effectiveness in symptomatic and presymptomatic patients. The international RESTORE registry was initiated following the approval of OA to accumulate more information on the course and outcomes of OA in patients with SMA.

OA was approved in Japan in 2020 and marketed as gene replacement therapy for SMA from the same year. As a regulatory requirement, a Japanese all‐case post‐marketing surveillance (PMS) was required and data were incorporated into the RESTORE registry. Insights from the Japanese PMS are important considering the limited numbers of Japanese patients enrolled in prior trials. Furthermore, those studies focused on OA monotherapy. This PMS was conducted in a real‐world clinical environment, providing insight into the actual use and outcomes of OA therapy in Japanese patients, without the strict eligibility requirements inherent to clinical trials. The broad range of patients allowed us to examine the safety and effectiveness of OA across subsets of patients, including SMA type, phenotype, treatment pattern, and screening status under real‐world conditions.

## 2 Methods

### 2.1 Overview and Ethics

The design of the long‐term RESTORE registry (ClinicalTrials.gov registration number: NCT04174157) is described in more detail elsewhere (Methods S1) [21, 22]. The following provides a brief summary of the methods, including aspects related to the Japanese PMS. We report updated interim analysis of the all‐case surveillance, which registered all patients treated with OA following its approval in May 2020 until June 2023. It is planned to observe patients for up to 15 years. We present data collected through to the cutoff date of May 23, 2025.

The ethical aspects of the RESTORE registry have been described previously [21, 22] and summarized in the Methods S1.

### 2.2 Patients

Patients were included in the Japanese PMS if they had a genetic diagnosis of SMA, were treated with OA, and the parental/legal guardian signed written informed consent/assent. Any patients involved in clinical trials of investigational products were ineligible. Patients who were subsequently enrolled in clinical trials of investigational products for SMA were withdrawn from the registry. Patients were included in this analysis if they received OA for the approved indication “spinal muscular atrophy, but only in patients negative for anti‐adeno‐associated virus 9 (AAV9) antibodies.”

### 2.3 Outcomes

Data collected included baseline demographics and clinical characteristics, treatment characteristics, event‐free survival (EFS), patient evaluations (including motor milestones and motor function), and adverse events (AEs).

Treatment patterns were classified as monotherapy, add‐on, transient add‐on, combination with OA, bridging to OA, and switching to OA (Methods S1) [23].

Safety outcomes are defined in the Methods S1.

Effectiveness outcomes comprised EFS, Children's Hospital of Philadelphia Infant Test of Neuromuscular Disorders (CHOP INTEND), developmental motor milestones, Hammersmith Infant Neurological Examination Section 2 (HINE‐2), and Hammersmith Functional Motor Scale Expanded (HFMSE). Changes in HFMSE by ≥ 3 points, CHOP INTEND by ≥ 4 points, and HINE‐2 by ≥ 2 points were defined as minimum clinically important differences [22, 24, 25].

### 2.4 Data Analysis

Data were analyzed using summary statistics (mean, standard deviation [SD], median, minimum [min], maximum [max], number, percentage) as appropriate, as previously described [21, 22]. Other effectiveness outcomes were assessed and analyzed as previously reported [21, 22]. The effectiveness outcomes are described in more detail in the Methods S1.

## 3 Results

### 3.1 Patient Disposition

This analysis comprised 80 patients who had been registered and started treatment with OA at < 24 months old (mean of 11.0 months; Table 1, Table S1). Of these, 73 patients were still enrolled and being followed up. Seven patients had discontinued (one death, four were lost to follow‐up, and two entered another clinical trial). The patient who died was initially treated with risdiplam for 5 months, followed by a switch to OA, and then resumed risdiplam, which was continued until the day before the patient died.

**TABLE 1:** Patient characteristics and treatment history.

	Age at OA infusion					All patients
	< 3 months	≥ 3 to < 6 months	≥ 6 to < 12 months	≥ 12 to < 24 months	≥ 24 months^a^	
	(*N* = 14)	(*N* = 9)	(*N* = 22)	(*N* = 31)	(*N* = 4)	(*N* = 80)
Age at first symptom onset^b^	*n* = 9	*n* = 7	*n* = 20	*n* = 31	*n* = 4	*n* = 71
Mean ± SD, months	0.2 ± 0.4	0.7 ± 0.8	3.7 ± 2.8	5.9 ± 4.2	7.8 ± 7.6	4.2 ± 4.2
Median (min, max), months	0.0 (0, 1)	1.0 (0, 2)	4.0 (0, 9)	5.0 (0, 15)	6.5 (0, 18)	3.0 (0, 18)
Age category						
< 6 months	9 (100.0)	7 (100.0)	13 (65.0)	16 (51.6)	2 (50.0)	47 (66.2)
≥ 6 to < 12 months	—	—	7 (35.0)	12 (38.7)	1 (25.0)	20 (28.2)
≥ 12 to < 24 months	—	—	—	3 (9.7)	1 (25.0)	4 (5.6)
Age at start of treatment						
Mean ± SD, months	1.4 ± 0.8	2.4 ± 1.4	5.5 ± 3.4	12.4 ± 5.7	14.8 ± 10.2	7.6 ± 6.5
Median (min, max), months	2.0 (0.0, 2.0)	3.0 (0.0, 5.0)	6.0 (1.0, 11.0)	14.0 (1.0, 22.0)	16.5 (2.0, 24.0)	6.0 (0.0, 24.0)
Age at OA infusion						
Mean ± SD, months	1.6 ± 0.7	3.8 ± 0.8	8.4 ± 1.4	17.6 ± 3.5	24.0 ± 0.0	11.0 ± 7.4
Median (min, max), months	2.0 (0, 2)	4.0 (3, 5)	8.0 (6, 11)	17.0 (12, 23)	24.0 (24, 24)	10.0 (0, 24)
Weight						
At initial SMA diagnosis, mean ± SD, kg	4.0 ± 1.0	4.0 ± 1.0	6.4 ± 2.1	7.5 ± 1.6	7.6 ± 2.5	6.2 ± 2.2
At start of OA infusion, mean ± SD, kg	4.4 ± 1.1	5.1 ± 1.2	7.8 ± 1.0	8.5 ± 1.5	8.7 ± 1.3	7.2 ± 2.0
Duration of observation from OA infusion to cutoff date of May 2025						
Mean ± SD, months	39.9 ± 10.8	45.3 ± 11.7	39.7 ± 14.1	46.1 ± 10.9	57.1 ± 3.3	43.7 ± 12.3
Median (min, max), months	38.9 (28.3, 58.5)	42.6 (29.5, 59.6)	34.8 (15.8, 59.4)	47.3 (12.6, 59.5)	58.0 (52.5, 59.9)	43.1 (12.6, 59.9)
Sex						
Female	4 (28.6)	4 (44.4)	8 (36.4)	19 (61.3)	3 (75.0)	38 (47.5)
Male	10 (71.4)	5 (55.6)	14 (63.6)	12 (38.7)	1 (25.0)	42 (52.5)
SMA type^b^, ^c^	*n* = 7	*n* = 7	*n* = 20	*n* = 31	*n* = 4	*n* = 69
1	7 (100.0)	7 (100.0)	17 (85.0)	21 (67.7)	2 (50.0)	54 (78.3)
2	0	0	3 (15.0)	10 (32.3)	2 (50.0)	15 (21.7)
Screened for SMA as a newborn						
Yes	5 (35.7)	3 (33.3)	2 (9.1)	0	0	10 (12.5)
No	9 (64.3)	6 (66.7)	20 (90.9)	31 (100.0)	4 (100.0)	70 (87.5)
Symptomatic at the time of SMA diagnosis						
Yes	8 (57.1)	7 (77.8)	20 (90.9)	31 (100.0)	4 (100.0)	70 (87.5)
No	6 (42.9)	2 (22.2)	2 (9.1)	0	0	10 (12.5)
SMA symptoms at SMA diagnosis^b^, ^d^	*n* = 8	*n* = 7	*n* = 20	*n* = 31	*n* = 4	*n* = 70
Hypotonia	8 (100.0)	7 (100.0)	19 (95.0)	31 (100.0)	4 (100.0)	69 (98.6)
Limb weakness	8 (100.0)	5 (71.4)	20 (100.0)	27 (87.1)	4 (100.0)	64 (91.4)
Pneumonia or respiratory symptoms	4 (50.0)	4 (57.1)	5 (25.0)	6 (19.4)	1 (25.0)	20 (28.6)
Tongue fasciculations	3 (37.5)	5 (71.4)	15 (75.0)	15 (48.4)	0	38 (54.3)
Developmental delay	2 (25.0)	2 (28.6)	11 (55.0)	22 (71.0)	2 (50.0)	39 (55.7)
Constipation	1 (12.5)	1 (14.3)	2 (10.0)	5 (16.1)	0	9 (12.9)
Swallowing or feeding difficulties	4 (50.0)	3 (42.9)	6 (30.0)	7 (22.6)	1 (25.0)	21 (30.0)
Number of copies of the *SMN2* gene^e^						
Two	10 (71.4)	6 (66.7)	11 (50.0)	12 (38.7)	1 (25.0)	40 (50.0)
Three	4 (28.6)	3 (33.3)	11 (50.0)	19 (61.3)	3 (75.0)	40 (50.0)
SMA function status at first dose of OA^f^	*n* = 13	*n* = 9	*n* = 22	*n* = 31	*n* = 4	*n* = 79
Non‐sitter	13 (100.0)	9 (100.0)	22 (100.0)	22 (71.0)	2 (50.0)	68 (86.1)
Sitter	0	0	0	9 (29.0)	1 (25.0)	10 (12.7)
Standing	0	0	0	0	1 (25.0)	1 (1.3)
History of tracheostomy	1	1	2	0	0	4
Treatment courses						
OA infusion monotherapy	6 (42.9)	0	5 (22.7)	12 (38.7)	1 (25.0)	24 (30.0)
Add‐on^g^	1 (7.1)	1 (11.1)	0	0	0	2 (2.5)
Transient add‐on^h^	1 (7.1)	0	0	0	0	1 (1.3)
Combination with OA infusion^i^	2 (14.3)	4 (44.4)	2 (9.1)	2 (6.5)	0	10 (12.5)
Bridge to OA infusion^j^	4 (28.6)	4 (44.4)	12 (54.5)	7 (22.6)	1 (25.0)	28 (35.0)
Switch to OA infusion^k^	0	0	3 (13.6)	10 (32.3)	2 (50.0)	15 (18.8)
Primary reason for switching to OA infusion	*n* = 6	*n* = 8	*n* = 17	*n* = 19	*n* = 3	*n* = 53
Perceived lack of drug effect^l^	1 (16.7)	0	1 (5.9)	0	0	2 (3.8)
Parent's/caregiver's/patient's decision	4 (66.7)	6 (75.0)	13 (76.5)	14 (73.7)	2 (66.7)	39 (73.6)
Alternative treatment available and reimbursed	1 (16.7)	2 (25.0)	3 (17.6)	5 (26.3)	1 (33.3)	12 (22.6)

*Note:* Values are *n* (%) of patients unless otherwise stated.

Abbreviations: max, maximum; min, minimum; OA, onasemnogene abeparvovec; SD, standard deviation; SMA, spinal muscular atrophy; *SMN2*, survival motor neuron 2.

^a^ Four patients were ≥ 24 months old. Although the data includes results for patients aged ≥ 24 months at the time of OA infusion due to removal of private information (month and date of birth) used to calculate age, it was confirmed that OA was administered at < 24 months of age in all patients.

^b^ Includes only patients with symptoms.

^c^ There were no patients with SMA type 0, 3 or 4.

^d^ Patient may have more than one response.

^e^ There were no patients with one or four or more copies of the *SMN2* gene.

^f^ None of the patients were walkers. One patient did not have information for functional status.

^g^ Treatment with nusinersen or risdiplam for the first time after OA infusion.

^h^ Nusinersen or risdiplam were administered once after OA infusion and discontinued subsequently.

^i^ Treatment with nusinersen or risdiplam, followed by OA, and resumption of nusinersen or risdiplam.

^j^ Switch to OA after short‐term treatment with nusinersen or risdiplam.

^k^ Switch to OA after long‐term treatment with nusinersen or risdiplam.

^l^ Based on the physician's subjective assessment.

By age at the time of OA infusion, 14 patients were < 3 months old, nine patients were ≥ 3 to < 6 months old, 22 patients were ≥ 6 to < 12 months old, and 31 patients were ≥ 12 to < 24 months old (Table S1). Most of the patients (76/80) had been followed up for > 2 years at the data cutoff.

### 3.2 Patient Characteristics

Table 1 presents the demographics and baseline clinical characteristics for all patients and by age at the time of OA infusion. The median (min, max) age was 3.0 (0, 18) months at the time of symptom onset, 6.0 (0.0, 24.0) months at the start of SMA treatment (any therapy), and 10.0 (0, 24) months at the time of OA infusion. Since OA infusion, the mean ± SD duration of follow‐up was 43.7 ± 12.3 months (median [min, max]: 43.1 [12.6, 59.9] months).

Fifty‐four patients (78.3%) were diagnosed with type 1 SMA. Only 10 (12.5%) patients were diagnosed following NBS. None received a genetic diagnosis of SMA prior to birth. Seventy patients (87.5%) had symptoms at the time of diagnosis; the two most common symptoms being hypotonia (98.6%) and limb weakness (91.4%). The number of *SMN2* gene copies was determined in all patients, and 40 patients each had two (50.0%) or three (50.0%) copies. Genetic tests revealed that 96.3% of patients were homozygous for exon 7 deletion (or exon 7 + exon 8) of *SMN1*, and 3.8% were heterozygous for deletion of exon 7 and/or 8 and subtle mutations. A test for the c.859G>C mutation in the *SMN2* gene (a positive modifier associated with a milder phenotype [26, 27]) was performed in 12 patients, and all 12 were negative.

Among patients whose functional status was recorded at the start of OA treatment, all 44 patients aged < 12 months were non‐sitters. Of 31 patients aged ≥ 12 to < 24 months, 9 (29.0%) were sitters. Of 4 patients aged ≥ 24 months, one (25.0%) was a sitter and one (25.0%) could stand (Table 1). This classification shows the functional status at the time of OA infusion based on the evaluation by the attending physician. It should be noted that, of the 44 non‐sitters aged < 12 months, 13 were aged < 3 months and were therefore not old enough to demonstrate this milestone at the start of treatment. Accordingly, it may not indicate an appropriate status relative to the actual developmental state of the infants.

Twelve patients (15.2%) had an immediate family history of SMA.

### 3.3 Treatment Characteristics

Table 1 summarizes the treatment characteristics overall and by age at OA infusion. The treatment modalities were bridge to OA infusion in 28 (35.0%), OA infusion monotherapy in 24 (30.0%), switch to OA infusion in 15 (18.8%), combination with OA infusion in 10 (12.5%), add‐on of nusinersen or risdiplam in two (2.5%), and transient add‐on in one (1.3%). Forty‐seven patients had ever‐received nusinersen and 16 had ever‐received risdiplam; the characteristics of these patients are presented in Tables S2 and S3, respectively, with stratification by number of *SMN2* gene copies. The primary reasons for switching to OA infusion were the parent's/caregiver's/patient's decision in 39 (73.6%), and availability and reimbursement as an alternative treatment in 12 (22.6%). The characteristics of patients according to the therapeutic modality are presented in Table S4.

### 3.4 Characteristics of Patients Diagnosed With SMA Following NBS

Of 10 patients diagnosed with SMA following NBS (Table S5), three had symptoms at diagnosis (hypotonia with other symptoms) and four had symptoms at enrollment in the PMS. Four patients had two copies and the others had three *SMN2* gene copies. The treatment course involved OA infusion monotherapy in four patients, bridge to OA infusion in three patients, combination with OA infusion in two patients, and switch to OA infusion in one patient. The interval between genetic confirmation of SMA and start of therapy ranged from 0 to 9 days for nusinersen/risdiplam and from 10 to 246 days for OA.

### 3.5 Safety

Treatment‐emergent adverse events (TEAEs) were reported in all 80 patients (100.0%), with grade ≥ 3 TEAEs in 65 (81.3%) and serious TEAEs in 55 (68.8%) (Table 2). TEAEs considered related to OA infusion occurred in 79 (98.8%), and were considered serious in 21 (26.3%). Adverse events of special interest (AESI) were reported in 74 patients (92.5%). Seventy‐one patients (88.8%) experienced AESI ≤ 2 weeks after OA infusion. The most common types of AESI were hepatotoxicity (72, 90.0%), transient thrombocytopenia (50, 62.5%), cardiac AEs (27, 33.8%), and new incidence of hematological disorders (13, 16.3%). Thrombotic microangiopathy (four, 5.0%), new incidence of neurological disorders (three, 3.8%), and new incidence of autoimmune disorders (one, 1.3%) were rare. There were no cases with signs or symptoms that may be suggestive of dorsal root ganglia toxicity or new malignancies. Among 21 patients with serious TEAEs, 20 patients had at least one AESI, of which 16 were hospitalized because of the AESI.

**TABLE 2:** TEAEs and AESI by age at OA infusion.

	Age at OA infusion					All patients
	< 3 months	≥ 3 to < 6 months	≥ 6 to < 12 months	≥ 12 to < 24 months	≥ 24 months^a^	
	(*N* = 14)	(*N* = 9)	(*N* = 22)	(*N* = 31)	(*N* = 4)	(*N* = 80)
≥ 1 TEAE (any grade)	14 (100.0)	9 (100.0)	22 (100.0)	31 (100.0)	4 (100.0)	80 (100.0)
Grade ≥ 3 TEAEs	7 (50.0)	8 (88.9)	19 (86.4)	27 (87.1)	4 (100.0)	65 (81.3)
Any serious TEAEs	9 (64.3)	8 (88.9)	15 (68.2)	21 (67.7)	2 (50.0)	55 (68.8)
TEAEs related to OA infusion	13 (92.9)	9 (100.0)	22 (100.0)	31 (100.0)	4 (100.0)	79 (98.8)
Serious TEAEs related to OA infusion	2 (14.3)	2 (22.2)	5 (22.7)	12 (38.7)	0	21 (26.3)
Any AESI	11 (78.6)	8 (88.9)	20 (90.9)	31 (100.0)	4 (100.0)	74 (92.5)
AESI according to time of onset after OA infusion						
≤ 2 weeks	11 (78.6)	7 (77.8)	19 (86.4)	30 (96.8)	4 (100.0)	71 (88.8)
> 2 to ≤ 4 weeks	1 (7.1)	1 (11.1)	5 (22.7)	6 (19.4)	1 (25.0)	14 (17.5)
> 4 weeks to ≤ 3 months	4 (28.6)	2 (22.2)	3 (13.6)	8 (25.8)	3 (75.0)	20 (25.0)
> 3 to ≤ 6 months	1 (7.1)	1 (11.1)	0	1 (3.2)	0	3 (3.8)
> 6 to ≤ 12 months	1 (7.1)	0	1 (4.5)	2 (6.5)	0	4 (5.0)
> 12 to ≤ 24 months	2 (14.3)	1 (11.1)	1 (4.5)	0	0	4 (5.0)
> 24 months	0	0	0	0	1 (25.0)	1 (1.3)
Categories of AESI						
Hepatotoxicity	11 (78.6)	8 (88.9)	19 (86.4)	30 (96.8)	4 (100.0)	72 (90.0)
Transient thrombocytopenia	3 (21.4)	6 (66.7)	12 (54.5)	25 (80.6)	4 (100.0)	50 (62.5)
Thrombotic microangiopathy	0	0	0	4 (12.9)	0	4 (5.0)
Cardiac AEs	6 (42.9)	4 (44.4)	6 (27.3)	8 (25.8)	3 (75.0)	27 (33.8)
Signs or symptoms that may be suggestive of dorsal root ganglia toxicity	0	0	0	0	0	0
New malignancies	0	0	0	0	0	0
New incidence of neurological disorders^b^	1 (7.1)	0	1 (4.5)	1 (3.2)	0	3 (3.8)
New incidence of autoimmune disorders^c^	0	0	0	1 (3.2)	0	1 (1.3)
New incidence of hematological disorders^d^	0	1 (11.1)	2 (9.1)	9 (29.0)	1 (25.0)	13 (16.3)

*Note:* Values are *n* (%) of patients.

Abbreviations: AE, adverse event; AESI, adverse event of special interest; OA, onasemnogene abeparvovec; TEAE, treatment‐emergent adverse event.

^a^ Four patients were ≥ 24 months old. Although the data includes results for patients aged ≥ 24 months at the time of OA infusion due to removal of private information (month and date of birth) used to calculate age, it was confirmed that OA was administered at < 24 months of age in all patients.

^b^ Comprised cerebral atrophy, motor developmental delay, and seizure in one patient each.

^c^ Comprised hypersensitivity in one patient.

^d^ Comprised cardio‐respiratory arrest, troponin T increased, troponin I increased, blood pressure increased, bradycardia, blood creatine phosphokinase increased, arrhythmia, thrombotic microangiopathy, nocturnal dyspnea, hepatomegaly, cardiac failure congestive, hypertension, N‐terminal prohormone brain natriuretic peptide increased in one patient each.

The frequencies of some AESI varied by age at OA infusion. In particular, the frequencies of hepatotoxicity and transient thrombocytopenia tended to be lower among patients aged < 12 months than in patients aged ≥ 12 months, and the rates were numerically lowest in patients aged < 3 months. By age group at OA infusion, the respective frequencies of any AESI, hepatotoxicity, and transient thrombocytopenia were as follows: age < 3 months (*n* = 14)—78.6%, 78.6%, and 21.4%; age ≥ 3 to < 6 months (*n* = 9)—88.9%, 88.9%, and 66.7%; age ≥ 6 to < 12 months (*n* = 22)—90.9%, 86.4%, and 54.5%; age ≥ 12 to < 24 months (*n* = 31)—100.0%, 96.8%, and 80.6%; age ≥ 24 months (*n* = 4)—100.0%, 100.0%, and 100.0%.

The frequencies of TEAEs according to System Organ Class and Preferred Term are shown in Table S6 among all patients and by age at OA infusion. The five most common TEAEs overall were pyrexia (81.3%), aspartate aminotransferase increased (66.3%), alanine aminotransferase increased (65.0%), platelet count decreased (50.0%), and vomiting (40.0%). Most categories of TEAEs, particularly blood and lymphatic system disorders (e.g., thrombocytopenia and thrombotic microangiopathy), gastrointestinal disorders (e.g., vomiting and nausea), general disorders and administration site conditions (e.g., pyrexia), and infections and infestations (e.g., pneumonia and bronchitis) either did not occur or were less frequent in patients aged < 3 months at OA infusion than in older patients.

In terms of the timing of the first TEAEs after OA infusion, most patients experienced TEAEs within the early period (≤ 2 weeks after OA infusion). For example, thrombocytopenia, thrombotic microangiopathy, and nausea were reported in 11.3%, 5.0%, and 6.3% of patients ≤ 2 weeks after OA infusion, but not at later times. However, TEAEs related to infections and infestations were more frequent in the later period, with frequency increasing from 3.8% in ≤ 2 weeks to ~20% from 6 months onwards (Table S7).

A glucocorticoid was administered to all 80 patients (100.0%). The weighted average mean ± SD prednisolone dose was 0.8 ± 0.2 (min, max: 0.5, 1.5) mg/kg/day and the mean ± SD duration of prednisolone administration was 2.9 ± 1.2 months (median [min, max]: 2.69 [1.0, 7.5] months).

One patient with type 1 SMA (2 months old at diagnosis, 7 months old at OA infusion, two *SMN2* gene copies) who received OA in combination with risdiplam died due to respiratory failure. This patient experienced cardiopulmonary arrest 482 days after OA infusion and died the same day. A causal relationship to OA was ruled out.

Tracheostomy was reported after initial SMA therapy in 11 patients (Table S8). Among these, tracheostomy was performed before OA infusion in four patients (bridge to OA in two patients and switch to OA in two patients). The other seven patients underwent tracheostomy sometime after OA infusion due to pneumonia in three patients, disease progression without an acute cause in two patients, and respiratory failure and upper respiratory illness in one patient each.

### 3.6 Effectiveness

Seventy‐nine patients were alive and included in effectiveness analyzes. For four patients lost to follow‐up and two patients who entered clinical trials, their effectiveness outcomes were analyzed through to their last known follow‐up.

#### 3.6.1 Event‐Free Survival

The 3‐year EFS, defined as the avoidance of death or the requirement of permanent ventilatory support, was 88.4% since first treatment and 93.0% since OA administration.

#### 3.6.2 Motor Milestones

**FIGURE 1:**
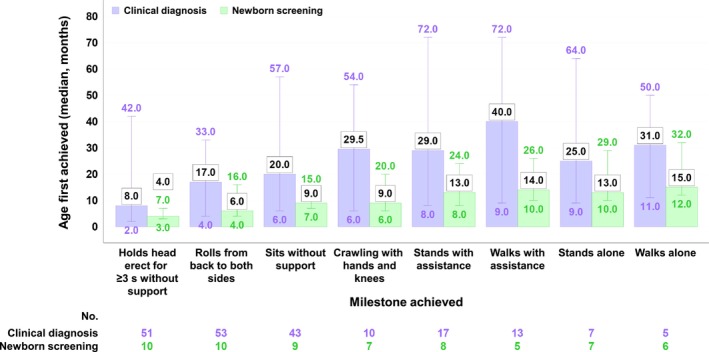
Median age of milestone achievement in patients diagnosed with SMA based on clinical diagnosis or newborn screening. The figure only shows data for patients whose age was recorded at the time the milestone was achieved. Error bars indicate the minimum and maximum values. SMA, spinal muscular atrophy.

Among 39 patients with two or more developmental motor milestone assessments, 25 (64.1%) achieved new milestones and six (15.4%) maintained milestones. Eight patients (20.5%) did not achieve new milestones and none showed loss of milestones. Figure 1 shows the median age at which the first milestone was achieved in patients divided according to diagnosis route. All milestones were consistently achieved 4 to 26 months earlier in patients diagnosed following NBS than in patients with clinical diagnosis. When patients with two or more developmental motor milestone assessments were stratified by the number of *SMN2* gene copies, out of 25 evaluable patients with two *SMN2* copies, 15 (60.0%) achieved additional developmental milestones, two (8.0%) maintained their milestones, and eight (32.0%) did not achieve motor milestones. Among 14 patients with three *SMN2* copies, 10 (71.4%) achieved additional developmental milestones and four (28.6%) maintained their milestones.

#### 3.6.3 CHOP INTEND

**FIGURE 2:**
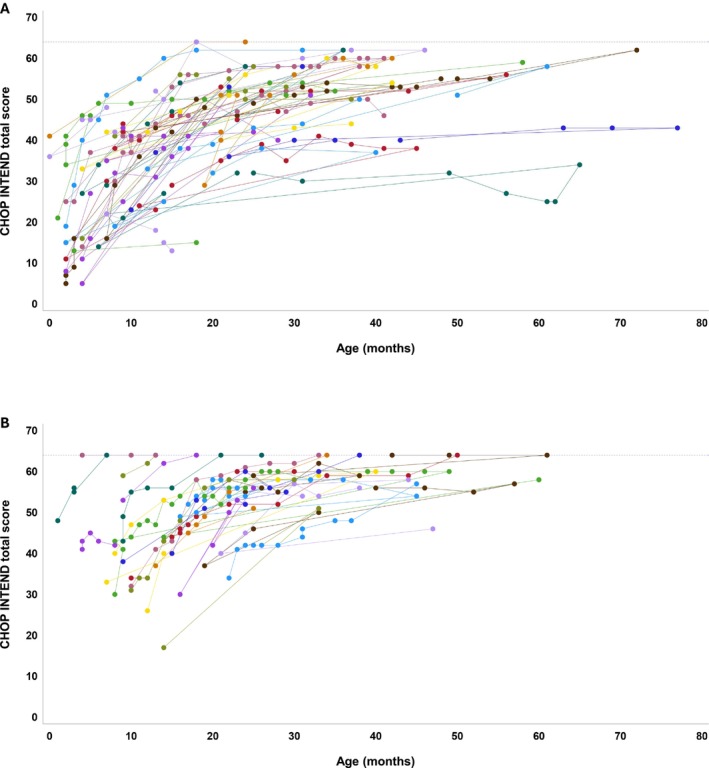
Spaghetti chart of CHOP INTEND total scores over age in patients with two copies (A) or three copies (B) of the *SMN2* gene. The dashed line indicates the maximum score (64 points). Data are shown for patients with at least two assessments of CHOP INTEND. CHOP INTEND scores were available for 35 of 40 patients with two copies of the *SMN2* gene and in 31 of 40 patients with three copies of the *SMN2* gene. CHOP INTEND was assessed once in one and three patients, respectively, and was not assessed in four and six patients, respectively. CHOP INTEND, Children's Hospital of Philadelphia Infant Test of Neuromuscular Disorders.

CHOP INTEND scores were available for 66 patients (35 with two and 31 with three *SMN2* gene copies). The total score increased or remained stable over time in the majority of patients with two or three *SMN2* gene copies (Figure 2A,B). Among patients with two *SMN2* gene copies, the total score increased by ≥ 4 points in 29 of 35 evaluable patients (82.9%), and 29 (82.9%) achieved a total score of ≥ 40 points (mean ± SD interval between first and last assessments: 28.0 ± 15.3 months). Of 31 evaluable patients with three *SMN2* gene copies, the total score increased by ≥ 4 points in 25 patients (80.6%), and 30 (96.8%) achieved a total score of ≥ 40 points (mean ± SD interval: 18.0 ± 13.0 months). Among four patients diagnosed by NBS with available data, the CHOP INTEND total score increased by ≥ 4 points in three patients (75.0%), and all four (100.0%) achieved a total score of ≥ 40 points.

In 56 patients with at least two assessments ≥ 6 months apart, 30 (53.6%) achieved a total score of ≥ 40 points, 20 (35.7%) maintained a total score of ≥ 40 points, and six (10.7%) did not achieve a total score of ≥ 40 points (Table 3). Table 3 also shows achievement of CHOP INTEND scores among patients stratified by NBS and clinical diagnosis. Data were available for one patient diagnosed following NBS; this patient achieved a score of ≥ 40 points. Results for patients with clinical diagnosis are similar to those of all patients with available data.

**TABLE 3:** Achievement of CHOP INTEND total scores of ≥ 40 according to number of copies of the *SMN2* gene and diagnostic method^a^.

	Copies of *SMN2* gene		Diagnostic method		All patients
	Two copies	Three copies	Newborn screening	Clinical diagnosis	
	(*N* = 32)	(*N* = 24)	(*N* = 1)	(*N* = 55)	(*N* = 56)
Achieved score of ≥ 40 points	20 (62.5)	10 (41.7)	1 (100.0)	29 (52.7)	30 (53.6)
Maintained score of ≥ 40 points	6 (18.8)	14 (58.3)	0	20 (36.4)	20 (35.7)
Did not achieve a score of ≥ 40 points^b^	6 (18.8)	0	0	6 (10.9)	6 (10.7)

*Note:* Values are *n* (%) of patients.

Abbreviations: CHOP INTEND, Children's Hospital of Philadelphia Infant Test of Neuromuscular Disorders; *SMN2*, survival motor neuron 2.

^a^ The analysis comprised patients treated with any therapy (not best‐supported care) with an interval of ≥ 6 months between the first and last assessment, and at least one assessment post‐initial therapy.

^b^ Includes patients with an initial score of ≥ 40 points and a final score of < 40 points.

#### 3.6.4 HINE‐2

Among 20 patients with at least two HINE‐2 assessments, the HINE‐2 total score increased by ≥ 2 points from the initial assessment to the final assessment in 18 patients overall (90.0%). When divided by diagnostic method, this score increased by ≥ 2 points in two of two patients (100.0%) diagnosed following NBS and in 16 of 18 patients (88.9%) with clinical diagnosis (Table S9). Furthermore, the scores increased by ≥ 2 points in three of three patients (100.0%) without symptoms at diagnosis, in 15 of 17 patients (88.2%) with symptoms at diagnosis (Table S10), in 10 of 11 patients (90.9%) with two *SMN2* gene copies, and in eight of nine patients (88.9%) with three *SMN2* gene copies (Table S11).

#### 3.6.5 HFMSE

Among 30 patients with at least two assessments of HFMSE, the HFMSE total score increased by ≥ 3 points in 25 patients (83.3%) and decreased by ≥ 3 points in one patient (3.3%). According to the diagnosis method (Table S9), the score was maintained in one of one patient (100.0%) diagnosed after NBS. Among four patients without symptoms at diagnosis (Table S10), the score increased by ≥ 3 points in two patients (50.0%) and was maintained in two patients (50.0%). Of 26 patients with symptoms at diagnosis, the score increased by ≥ 3 points in 23 patients (88.5%) and decreased by ≥ 3 points in one patient (3.8%). Of 12 patients with two *SMN2* gene copies (Table S11), the HFMSE score increased by ≥ 3 points in 11 patients (91.7%). Of 18 patients with three *SMN2* gene copies (Table S11), the score increased in 14 patients (77.8%) and decreased in one patient (5.6%).

## 4 Discussion

In this article, we presented the results of a 5‐year interim analysis of the Japanese PMS for OA, as part of the RESTORE registry. To our knowledge, this is one of the largest real‐world surveys of *SMN1* gene replacement therapy for pediatric patients with SMA in Japan, comprising 80 patients, including 10 who were diagnosed following NBS and subsequently received OA. Notably, most patients had been followed up for ≥ 2 years after OA treatment (76 patients; 95.0%), and most patients were still enrolled (73 patients; 91.3%).

### 4.1 Safety

The safety profile of OA in Japanese patients treated with OA was broadly consistent with that reported in the interim analysis of the overall RESTORE registry [22], prior clinical trials [7, 9, 10, 11, 12, 28, 29, 30, 31], and real‐world studies [13, 14, 16, 17, 18, 19, 32, 33, 34]. However, some AESI (notably hepatotoxicity and transient thrombocytopenia) occurred at a greater frequency in the Japanese population than in the overall RESTORE population [22].

Although the reason(s) for the higher frequency of certain AESI are not fully clear, possible contributing factors may be the lower NBS rate (12.5% vs. 58.3%) and the older age at OA infusion (mean: 11.0 vs. 6.38 months) in this Japanese PMS than in the prior analysis of the RESTORE registry, as well as the greater dose of OA in relation to the greater body weight in these older patients [22]. The prior report of RESTORE was limited to OA monotherapy only and the difference of treatment pattern should be considered. Genetic factors particular to Japanese ethnicity may also predispose to a higher risk of AEs, but this is speculative. Also, AE data around the time of OA infusion were unavailable for some patients enrolled after administration and retrospective data collection was not possible. Thus, the peri‐infusion AE rates may be underestimated in the overall RESTORE population.

Administration in the neonatal period—ideally in the presymptomatic stage following NBS—might coincide with a period of greater immune tolerance [35, 36] and consequently reduce the risk of immune‐related AESI such as hepatotoxicity and thrombocytopenia. Indeed, the frequencies of many categories of TEAEs were lower in patients who received OA infusion at < 3 months old than in older patients.

Notably, glucocorticoids were used in all patients to control AEs, and prophylactic use is recommended for the prevention of liver dysfunction [37]. The median duration of administration was 2.69 months (mean: 2.9 months), which is broadly similar to that in other countries (around 85 days) [38, 39]. However, the mean dose of prednisolone (weighted average: 0.8 ± 0.2 mg/kg/day; min, max: 0.5, 1.5 mg/kg/day) was slightly lower than the dose recommended in the package insert (1.0 mg/kg/day). This may reflect the prednisolone reduction period because we calculated the weighted average prednisolone dose, which was adjusted by the total duration of prednisolone treatment in individual patients. Hepatic disorders, thrombocytopenia, and thrombotic microangiopathy were reported and are listed as precautions in the package insert [40]. These types of AEs may be class effects of gene therapy involving the patient's immune response to AAV9, in particular [34, 41]. Beyond prophylactic prednisolone use for preventing liver dysfunction, patients may also require other treatments to manage AEs, such as a complement inhibitor to treat thrombotic microangiopathy [42, 43]. Thus, careful observation and management are required to prevent potentially serious complications or to detect and promptly treat such immune‐related events.

Considering the patterns of AEs by age group and comparing our data with those of other studies, we believe earlier *SMN1* replacement therapy could be warranted in patients identified by NBS and in younger age groups, especially those with early onset of symptoms. This may apply to current treatment recommendations not only in efficacy but also in safety, stating “In newly diagnosed patients, including those identified by NBS, any delay of treatment should be avoided. Ideally, the time frame between diagnosis and initiation of a disease‐modifying treatment should be the shortest possible” [44].

Overall, our findings further demonstrate the need for careful monitoring of patients treated with OA, particularly in the early stage of administration because most patients experienced TEAEs within 2 weeks after OA infusion. Although no clinically significant late TEAEs were observed, further long‐term follow‐up and continued observation remain important to monitor potential new safety signals after treatment.

### 4.2 Effectiveness of OA

OA infusion demonstrated effectiveness across a range of assessments, including motor milestones, CHOP INTEND, HINE‐2, and HFMSE. For example, out of 39 patients with two or more developmental milestones, 25 achieved new motor milestones, six maintained their milestones, and eight did not develop new milestones. Nevertheless, none showed a loss of motor milestones. Additionally, the CHOP INTEND total scores increased to ≥ 40 points in 82.9% of evaluable patients, and 82.9% of patients had an increase of ≥ 4 points. Additionally, improvements in HINE‐2 and HFMSE were also observed: the HINE‐2 score increased by ≥ 2 points in 90.0% of evaluable patients and the HFMSE total score increased by ≥ 3 points in 83.3% of evaluable patients. However, these outcomes were assessed in fewer patients.

The PMS included 24 patients who received OA as monotherapy, while the majority of the other patients received OA as part of combination therapy (10 patients), as a bridge to OA (28 patients), or were switched to OA (15 patients). Bridging with short‐term use or switching after long‐term use of other drugs was reported in a prior real‐world study in Europe [20]. Importantly, the motor function changes following OA infusion appeared to be independent of the treatment pattern.

### 4.3 Role of NBS and Effectiveness of OA in Patients Identified by NBS

As mentioned above, only a small proportion of patients underwent NBS in this PMS (12.5% vs. 58.3% in the overall RESTORE registry), reflecting Japanese clinical practice at the time when OA was the first product approved for presymptomatic SMA in Japan. Many of the patients enrolled in this PMS were diagnosed with SMA before a nationwide NBS program had been implemented, and NBS was subsidized by a handful of local governments, limiting its use in clinical practice. However, NBS is now becoming part of mainstream clinical practice in Japan [45]. As highlighted in a recent expert consensus, “SMA should be included in newborn screening programs in countries where at least one disease‐modifying treatment is readily available. Patients identified by NBS should be evaluated by a pediatric neurologist experienced with neuromuscular diseases as soon as possible” [44, 46]. Furthermore, more favorable outcomes of OA can be achieved in at‐risk presymptomatic infants who are treated with OA within 6 weeks of birth [11, 12]. However, some patients identified by NBS may already be symptomatic, and their outcomes after receiving disease‐modifying therapy including OA may not match those of presymptomatic patients [44]. Thus, gene replacement therapy should be initiated as early as possible not only in symptomatic patients but also in newly diagnosed presymptomatic patients. All patients in this PMS who were diagnosed by NBS received their initial treatment within 23 days of genetic confirmation, including those who received OA monotherapy. Although three patients received OA 115–246 days after genetic confirmation, this likely represents the treatments available at the time of diagnosis, the timing of subsequent approval of OA, and delayed OA initiation due to clinical factors (e.g., maternally transferred AAV9 antibodies). It is anticipated that OA will be considered as a treatment option sooner after genetic diagnosis following NBS in the future.

Considering these factors, it is important to analyze the safety and effectiveness in patients with and without NBS separately. Unfortunately, the number of patients diagnosed following NBS was small (10 patients), limiting our ability to interpret the results in a meaningful way. Nevertheless, a notable finding is that motor milestones were achieved 4 to 26 months earlier in patients diagnosed by NBS than in patients diagnosed by clinical diagnosis (Figure 1). Prompt initiation of treatment after birth can help patients achieve motor milestones earlier, without marked developmental delays compared with individuals without SMA. Overall, our evidence further demonstrates the effectiveness of OA in presymptomatic patients who were identified following NBS. This evidence will be important in the future when we expect most cases of SMA to be detected by NBS in the presymptomatic stage, allowing prompt initiation of gene replacement therapy. However, we must acknowledge that some patients who were diagnosed with SMA following NBS received a disease‐modifying treatment before starting OA. Although no apparent difference in effectiveness was found among patients stratified by treatment modality in an earlier study [20], OA was not always administered close to the time of diagnosis. The interval between diagnosis and initiation of gene replacement therapy should be as short as possible, given the importance of preserving remaining motor neurons, and patients with type 1 SMA and/or two *SMN2* gene copies should be considered high priorities for urgent treatment [44].

### 4.4 Limitations

This report describes interim results with limited follow‐up, and continued monitoring of safety and effectiveness is needed; observation is expected to continue for up to 15 years. Some patients were missing data for changes in motor milestones, CHOP INTEND, HINE‐2, and HFMSE, either because the attending physician did not record the data or the assessments had not yet been done. Ongoing accumulation of these data will provide more insights into the long‐term effectiveness of OA. The registry was conducted as a single cohort, and no comparisons with other treatments were possible. Direct comparisons with registries in other countries or the overall RESTORE registry should be done cautiously due to differences in clinical practice, including whether SMA was diagnosed based on the presence of symptoms or NBS, and the use of other disease‐modifying treatments before OA infusion. The small number of patients diagnosed with SMA following NBS also prevented evaluation of differential outcomes in presymptomatic vs. symptomatic patients.

## 5 Conclusions

In conclusion, we have reported the findings of an all‐case PMS of *SMN1* one‐time gene replacement therapy for pediatric patients with SMA in Japan conducted in a real‐world clinical environment. The safety profile of OA infusion in Japanese patients with SMA was consistent with the previously reported safety profile. The real‐world findings suggest there were gains in or maintenance of motor milestones or motor function scores that were sustained over the observation period. These findings were apparent in patients with two or three *SMN2* gene copies. Ongoing follow‐up is expected to provide more insight into the value of early initiation of gene replacement therapy in patients with SMA, including in patients diagnosed following NBS.

## Author Contributions

As stipulated in the RESTORE bylaws, all publication topics were authored and approved by the members of the steering committee, and analyzes were performed by statisticians employed by the contract research organization managing the study data. All authors had access to the data, analyzed and interpreted the data, participated in the development and critical review of the manuscript, approved the final version of the manuscript for submission for publication, and are accountable for the accuracy and integrity of the work. Study conception and study design: K.S., R.S.F., K.B., K.T., K.Y., S.P.R., and F.K. Data acquisition: K.S., R.S.F., K.B., K.T., K.Y., I.T., S.P.R., and F.K. Data analysis: K.B. and F.K. Data interpretation: all authors. Drafting the manuscript: all authors. Critical review of the manuscript: all authors. Final approval: all authors. Accountable for the accuracy and integrity of the work: all authors.

## Funding

Novartis Gene Therapies Inc. funded the study, contributed to study design and the collection, analysis, and interpretation of data, and reviewed the manuscript. Novartis Pharma K.K. funded the publication and writing support through LESPEDEZA (a division of Omnicom Health Japan K.K.), contributed to data interpretation, and reviewed the manuscript.

## Conflicts of Interest

K.S. was a site principal investigator for Biogen, Novartis Gene Therapies Inc./Novartis, and Chugai/Roche clinical trials; has served on advisory boards for Novartis, Biogen, and Chugai/Roche; has received speakers' fees from Biogen, Novartis, and Chugai/Roche; and is a medical advisor for the Japan SMA Families Network. R.S.F. has received personal compensation for consulting and for advisory board participation from Novartis Gene Therapies Inc., Biogen, Novartis, Roche, and Scholar Rock; editorial fees from Elsevier for co‐editing a neurology textbook; license fees from the Children's Hospital of Philadelphia; and research funding from Novartis Gene Therapies Inc., Biogen, Roche/Genentech, and Scholar Rock. K.B., K.T., K.Y., I.T., H.K., S.P.R., and F.K. are employees of the Novartis group of companies or its affiliates and may hold stock or stock options in Novartis.

## Supporting information


Methods S1.
**Table S1:** Patient disposition.
**Table S2:** Treatment characteristics according to the number of copies of the *SMN2* gene among patients treated with nusinersen.
**Table S3:** Treatment characteristics according to the number of copies of the *SMN2* gene among patients treated with risdiplam.
**Table S4:** Treatment characteristics according to the therapeutic modality.
**Table S5:** Characteristics of patients diagnosed with SMA following newborn screening.
**Table S6:** TEAEs according to the age at OA infusion, by System Organ Class and Preferred Term (in ≥ 5% of patients).
**Table S7:** TEAEs according to the timing of the first event after OA infusion, by System Organ Class and Preferred Term (in ≥ 5% of patients).
**Table S8:** Characteristics of patients with tracheostomy (before or after OA infusion).
**Table S9:** Proportions of patients with changes in HFMSE and HINE‐2 scores according to newborn screening status.
**Table S10:** Proportions of patients with changes in HFMSE and HINE‐2 scores according to the presence of symptoms at diagnosis.
**Table S11:** Proportions of patients with changes in HFMSE and HINE‐2 scores according to the number of copies of the *SMN2* gene.

## Data Availability

The data sets generated and analyzed during the study are available from the RESTORE registry, as previously described [21, 22]. These data sets are not publicly available, but are available from the corresponding author/RESTORE steering committee on reasonable request.
