# Supplementary material for: Onasemnogene Abeparvovec in Patients With SMA: Interim Results of the RESTORE Registry in Japan
Source: Ann Clin Transl Neurol. 2026 Jun 9:10.1002/acn3.70437. Online ahead of print. doi: 10.1002/acn3.70437 (PMC13395036; doi:10.1002/acn3.70437)
Supplement: Supplementary file 1 — Methods S1. Table S1: Patient disposition. Table S2: Treatment characteristics according to the number of copies of the SMN2 gene among patients treated with nusinersen. Table S3: Treatment characteristics according to the number of copies of the SMN2 gene among patients treated with risdiplam. Table S4: Treatment characteristics according to the therapeutic modality. Table S5: Characteristics of patients diagnosed with SMA following newborn screening. Table S6: TEAEs according to the age at OA infusion, by System Organ Class and Preferred Term (in ≥ 5% of patients). Table S7: TEAEs according to the timing of the first event after OA infusion, by System Organ Class and Preferred Term (in ≥ 5% of patients). Table S8: Characteristics of patients with tracheostomy (before or after OA infusion). Table S9: Proportions of patients with changes in HFMSE and HINE‐2 scores according to newborn screening status. Table S10: Proportions of patients with changes in HFMSE and HINE‐2 scores according to the presence of symptoms at diagnosis. Table S11: Proportions of patients with changes in HFMSE and HINE‐2 scores according to the number of copies of the SMN2 gene. [file ACN3-9999-0-s001.pdf]

SUPPLEMENTARY MATERIALS

Onasemnogene Abeparvovec in Patients With SMA: Interim Results of the RESTORE Registry in Japan

Kayoko Saito, Kamal Benguerba, Ken Tsuchida, Kazushige Yazawa, Isao Tsumiyama, Hiromitsu Kayama, Sandra P. Reyna, Farid Khan, Richard S. Finkel

Corresponding author: Kayoko Saito, MD, PhD
E-mail: saito.kayoko@twmu.ac.jp

**Contents**	**Page**
**Methods S1.**	2
**TABLE S1.** Patient disposition.	5
**TABLE S2.** Treatment characteristics according to the number of copies of the *SMN2* gene among patients treated with nusinersen.	6
**TABLE S3.** Treatment characteristics according to the number of copies of the *SMN2* gene among patients treated with risdiplam.	7
**TABLE S4.** Treatment characteristics according to the therapeutic modality.	8
**TABLE S5.** Characteristics of patients diagnosed with SMA following newborn screening.	10
**TABLE S6.** TEAEs according to the age at OA infusion, by System Organ Class and Preferred Term (in ≥5% of patients).	11
**TABLE S7.** TEAEs according to the timing of the first event after OA infusion, by System Organ Class and Preferred Term (in ≥5% of patients).	13
**TABLE S8.** Characteristics of patients with tracheostomy (before or after OA infusion).	15
**TABLE S9.** Proportions of patients with changes in HFMSE and HINE-2 scores according to newborn screening status.	16
**TABLE S10.** Proportions of patients with changes in HFMSE and HINE-2 scores according to the presence of symptoms at diagnosis.	17
**TABLE S11.** Proportions of patients with changes in HFMSE and HINE-2 scores according to the number of copies of the *SMN2* gene.	18

Methods S1

Registry Overview

As previously described [1, 2], the RESTORE registry is governed by a steering committee of experts committed to ensuring data quality and data sharing. It is sponsored by Novartis Gene Therapies, Inc. (now part of Novartis Pharma AG), the manufacturer of onasemnogene abeparvovec (OA), and it was designed to monitor the history and treatment of spinal muscular atrophy (SMA), as well as the effectiveness and safety over a 15-year period in patients treated with disease-modifying treatments internationally. The registry is collecting data from the following sources: individual *de novo* clinical sites, data sharing agreements (e.g. with external academic registries), managed and expanded access programs, and post-marketing surveillance.

Ethical Aspects

The ethical aspects of the RESTORE registry have been described previously [1, 2]. The registry is being conducted in accordance with the ethical principles based on the Declaration of Helsinki, applicable laws on the protection of personal information, and national regulations, including the Ministerial Ordinance on Standards for Post-Marketing Surveillance and Testing of Regenerative Medicine Products (Ordinance of the Ministry of Health, Labour and Welfare No. 116 of 2017 and related notices), Good Pharmacoepidemiology Practice, and Strengthening the Reporting of Observational Studies in Epidemiology (STROBE) Guidelines [3]. All participating institutions obtained approval from the relevant Institutional Review Board or Independent Ethics Committee, and signed contracts with Novartis Pharma K.K. prior to patient registration.

The legal guardian and the patient (depending on their age) provided written informed consent and consented to the policy on the protection of personal information. With continuing follow-up, consent will be re-obtained from the patients when they reach an age at which they can provide informed consent.

Classification of Treatment Patterns

Treatment patterns were classified as monotherapy add-on, transient add-on, combination with OA, bridging to OA, and switching to OA [4]. In this classification, any treatment administered after OA is considered “add-on” if continued or as “transient add-on” if the additional treatment is discontinued. Combination treatments were defined as ongoing or addition of nusinersen and/or risdiplam after OA. Bridge to OA was defined as short-term treatment with nusinersen or

risdiplam (loading dose of nusinersen or treatment with risdiplam for ≤3 months). Switch to OA was defined as longer-term treatment with (≥1 maintenance dose of nusinersen or treatment with risdiplam for ≥3 months).

Safety Outcomes

Adverse events (AEs) were classified as treatment-emergent AEs (TEAEs) and AEs of special interest (AESI). AESI included hepatotoxicity, transient thrombocytopenia, thrombotic microangiopathy (TMA), cardiac events, signs and symptoms that may be suggestive of dorsal root ganglia toxicity, and delayed AEs regardless of the causal relationship with gene therapy (new onset of malignancies, neurological disorders, autoimmune diseases, or hematological disorders), which were defined using standardized medical terms in MedDRA, version 28.0 [1, 2]. The causality and severity of AEs were assessed by the attending physicians. All AEs were to be recorded for the first 12 months after the start of OA treatment. Serious AEs (including death), AESI, and non-serious AEs related to OA infusion were to be recorded for the remainder of the follow-up.

Data Analyses

Motor milestones were compared between the first and last assessments in patients with at least two assessments; at least one of the assessments had to be performed after OA treatment. Event-free survival, stratified by number of copies of the*SMN2* gene, was assessed using the Kaplan–Meier method and defined as the avoidance of death or the requirement of permanent ventilatory support. Permanent ventilatory support was defined as requiring either a tracheostomy or respiratory support for ≥16 hours per day (including non-invasive ventilatory support) continuously for ≥14 days in the absence of an acute reversible illness, excluding perioperative ventilation. Patients requiring permanent ventilatory support prior to treatment were excluded [2]. The prednisolone dose was calculated as the weighted average prednisolone dose, which was adjusted by the total duration of prednisolone treatment in individual patients. To protect personal information, if only the birth year was entered, the age in months was calculated from January 1st. Data analyses were performed using SAS version 9.4 (SAS Institute, Cary, NC, USA).

Supplemental References

1. R. S. Finkel, J. W. Day, D. C. De Vivo, et al., “RESTORE: A Prospective Multinational Registry of Patients With Genetically Confirmed Spinal Muscular Atrophy - Rationale and

Study Design," Journal of Neuromuscular Diseases 7 (2020): 145–152, https://doi.org/10.3233/jnd-190451.

2. L. Servais, J. W. Day, D. C. De Vivo, et al., "Real-World Outcomes in Patients With Spinal Muscular Atrophy Treated With Onasemnogene Abeparvovec Monotherapy: Findings From the RESTORE Registry," Journal of Neuromuscular Diseases 11 (2024): 425–442, https://doi.org/10.3233/jnd-230122.
3. J. P. Vandenbroucke, E. von Elm, D. G. Altman, et al. Strengthening the Reporting of Observational Studies in Epidemiology (STROBE): Explanation and Elaboration. Epidemiology 18 (2007): 805–835, https://doi.org/10.1097/ede.0b013e3181577511.
4. C. M. Proud, E. Mercuri, R. S. Finkel, et al., "Combination Disease-Modifying Treatment in Spinal Muscular Atrophy: A Proposed Classification," Annals of Clinical Translational Neurology 10 (2023): 2155–2160, https://doi.org/10.1002/acn3.51889.

TABLE S1. Patient disposition.

	Age at OA infusion					All patients
	<3 months (*N* = 14)	≥3 to <6 months (*N* = 9)	≥6 to <12 months (*N* = 22)	≥12 to <24 months (*N* = 31)	≥24 months [a] (*N* = 4)	(*N* = 80)
Still enrolled	14 (100.0)	9 (100.0)	20 (90.9)	26 (83.9)	4 (100.0)	73 (91.3)
Early termination/withdrawal	0	0	2 (9.1)	5 (16.1)	0	7 (8.8)
Death	0	0	1 (4.5)	0	0	1 (1.3)
Discontinued due to AE	0	0	0	0	0	0
Lost to follow-up	0	0	1 (4.5)	3 (9.7)	0	4 (5.0)
Withdrawal of consent	0	0	0	0	0	0
Physician's decision	0	0	0	0	0	0
Site terminated by sponsor	0	0	0	0	0	0
Registry is terminated by sponsor	0	0	0	0	0	0
Patient entered another clinical trial	0	0	0	2 (6.5)	0	2 (2.5)
Patients with follow-up visit	14 (100.0)	9 (100.0)	22 (100.0)	31 (100.0)	4 (100.0)	80 (100.0)
Enrolled for 1–2 years	2 (14.3)	0	2 (9.1)	0	0	4 (5.0)
Enrolled for 1–2 years with follow-up visit	2 (100.0)	0	2 (100.0)	0	0	4 (100.0)
Enrolled for >2 years	12 (85.7)	9 (100.0)	20 (90.9)	31 (100.0)	4 (100.0)	76 (95.0)
Enrolled for >2 years with follow-up visit	12 (100.0)	9 (100.0)	20 (100.0)	31 (100.0)	4 (100.0)	76 (100.0)

Note: Values are *n* (%) of patients.

Abbreviations: AE, adverse event; OA, onasemnogene abeparvovec.

[a] Four patients were ≥24 months old. Although the data includes results for patients aged ≥24 months at the time of OA infusion due to removal of private information (month and date of birth) used to calculate age, it was confirmed that OA was administered at <24 months of age in all patients.

TABLE S2. Treatment characteristics according to the number of copies of the *SMN2* gene among patients treated with nusinersen.

	**Two copies of *SMN2* (*N* = 28)**	**Three copies of *SMN2* (*N* = 19)**	**Total (*N* = 47)**
Interval from diagnosis to therapy			
Mean ± SD, months	0.7 ± 2.6	1.6 ± 3.8	1.1 ± 3.1
Median (min, max), months	0.3 (−2.4, 13.6)	0.3 (−0.3, 12.5)	0.3 (−2.4, 13.6)
Category			
0 to 6 months	26 (92.9)	16 (84.2)	42 (89.4)
>6 to 24 months	1 (3.6)	2 (10.5)	3 (6.4)
>24 months	0	0	0
Age at first dose			
Mean ± SD, months	3.9 ± 3.7	9.8 ± 6.9	6.3 ± 6.0
Median (min, max), months	3.0 (0, 15)	9.0 (0, 22)	5.0 (0, 22)
Age category			
<6 months	21 (75.0)	5 (26.3)	26 (55.3)
≥6 to <12 months	5 (17.9)	8 (42.1)	13 (27.7)
≥12 to <24 months	2 (7.1)	6 (31.6)	8 (17.0)
≥24 months	0	0	0
Duration of therapy			
Mean ± SD, months	6.7 ± 9.4	6.3 ± 11.5	6.6 ± 10.1
Median (min, max), months	2.1 (0.4, 45.0)	2.1 (0.4, 41.4)	2.1 (0.4, 45.0)

Note: Values are *n* (%) of patients unless otherwise stated.

Abbreviations: max, maximum; min, minimum; SD, standard deviation; *SMN2*, survival motor neuron 2.

TABLE S3. Treatment characteristics according to the number of copies of the *SMN2* gene among patients treated with risdiplam.

	Two copies of *SMN2* (*N* = 13)	Three copies of *SMN2* (*N* = 3)	Total (*N* = 16)
Interval from diagnosis to therapy			
Mean ± SD, months	17.7 ± 19.7	9.5 ± 15.9	16.2 ± 18.8
Median (min, max), months	7.9 (0.0, 59.5)	0.5 (0.0, 27.9)	7.2 (0.0, 59.5)
Category			
0 to 6 months	5 (38.5)	2 (66.7)	7 (43.8)
>6 to 24 months	3 (23.1)	0	3 (18.8)
>24 months	5 (38.5)	1 (33.3)	6 (37.5)
Age at first dose			
Mean ± SD, months	21.8 ± 19.6	16.7 ± 16.2	20.9 ± 18.6
Median (min, max), months	19.0 (2, 65)	14.0 (2, 34)	16.5 (2, 65)
Age category			
<6 months	3 (23.1)	1 (33.3)	4 (25.0)
≥6 to <12 months	3 (23.1)	0	3 (18.8)
≥12 to <24 months	1 (7.7)	1 (33.3)	2 (12.5)
≥24 months	6 (46.2)	1 (33.3)	7 (43.8)
Duration of therapy			
Mean ± SD, months	18.3 ± 13.3	12.5 ± 11.1	17.2 ± 12.8
Median (min, max), months	17.1 (0.6, 45.2)	13.5 (0.9, 23.1)	15.5 (0.6, 45.2)

Note: Values are *n* (%) of patients unless otherwise stated.

Abbreviations: max, maximum; min, minimum; SD, standard deviation; *SMN2*, survival motor neuron 2.

TABLE S4. Treatment characteristics according to the therapeutic modality.

	OA infusion		Transient add-on	Combination with OA infusion	Bridge to OA infusion	Switch to OA infusion	All patients
	Monotherapy (*N* = 24)	Add-on (*N* = 2)	(*N* = 1)	(*N* = 10)	(*N* = 28)	(*N* = 15) [a]	(*N* = 80) [b]
Age at OA infusion							
Mean ± SD, months	11.1 ± 6.8	3.5 ± 2.1	2.0	6.6 ± 5.2	9.6 ± 7.0	18.1 ± 5.8	11.0 ± 7.4
Median (min, max), months	12.5 (1, 24)	3.5 (2, 5)	2.0	4.0 (1, 15)	7.0 (0, 24)	19.0 (7, 24)	10.0 (0, 24)
Age category							
<6 months	6 (25.0)	2 (100.0)	1 (100.0)	6 (60.0)	8 (28.6)	0	23 (28.8)
≥6 to <12 months	5 (20.8)	0	0	2 (20.0)	12 (42.9)	3 (20.0)	22 (27.5)
≥12 to <24 months	12 (50.0)	0	0	2 (20.0)	7 (25.0)	10 (66.7)	31 (38.8)
≥24 months [c]	1 (4.2)	0	0	0	1 (3.6)	2 (13.3)	4 (5.0)
Time to therapy relative to SMA diagnosis							
Mean ± SD, months	0.9 ± 0.4	0.6 ± 0.0	0.5	3.1 ± 3.7	3.1 ± 3.1	13.1 ± 5.2	4.2 ± 5.4
Median (min, max), months	0.9 (0.3, 1.9)	0.6 (0.5, 0.6)	0.5	1.6 (0.4, 11.4)	1.6 (0.4, 12.7)	12.7 (5.0, 22.8)	1.4 (0.3, 22.8)
Time from diagnosis to treatment							
0 to ≤6 months	24 (100.0)	2 (100.0)	1 (100.0)	8 (80.0)	23 (82.1)	2 (13.3)	60 (75.0)
>6 to ≤24 months	0	0	0	2 (20.0)	5 (17.9)	13 (86.7)	20 (25.0)
>24 months	0	0	0	0	0	0	0
Primary reason for switching to OA infusion							
Perceived lack of drug effect	–	–	–	1 (10.0)	0	1 (6.7)	2 (3.8)
Motor function	–	–	–	1 (10.0)	0	0	1 (1.9)
Respiratory function	–	–	–	0	0	0	0
Swallowing or feeding ability for age	–	–	–	0	0	1 (6.7)	1 (1.9)
Electrophysiological/other biomarker response	–	–	–	0	0	0	0
No additional reason	–	–	–	0	0	0	0
AEs	–	–	–	0	0	0	0
Parent's/caregiver's/patient's decision	–	–	–	6 (60.0)	22 (78.6)	11 (73.3)	39 (73.6)

Alternative treatment available and reimbursed	–	–	–	3 (30.0)	6 (21.4)	3 (20.0)	12 (22.6)
Other	–	–	–	0	0	0	0
Duration since OA infusion							
Mean ± SD, months	43.8 ± 12.1	51.7 ± 0.2	58.5	44.0 ± 11.4	42.2 ± 11.7	44.2 ± 15.1	43.7 ± 12.3
Median (min, max), months	47.7 (12.6, 59.5)	51.7 (51.6, 51.8)	58.5	44.4 (28.8, 59.4)	38.9 (25.6, 59.6)	43.5 (15.8, 59.9)	43.1 (12.6, 59.9)
OA infusion exposure, person-year	87.6	8.6	4.9	36.6	98.4	55.2	291.4

Note: Values are *n* (%) of patients unless otherwise stated.

Abbreviations: AE, adverse event; max, maximum; min, minimum; OA, onasemnogene abeparvovec; SD, standard deviation; SMA, spinal muscular atrophy.

[a] Includes one patient who was initially treated with risdiplam for 5 months, followed by a switch to OA, and then resumed risdiplam, which was continued until the day before the patient died. This patient was counted as a switch to OA rather than combination with OA due to the definition of the treatment pattern in the statistical analysis plan.

[b] Nusinersen was added in 1 patient and risdiplam in 13 patients after OA infusion. The stated reason for additional administration of nusinersen was lack of efficacy (1 patient for motor function), and the stated reasons for additional administration of risdiplam were lack of efficacy (4 patients; 1 patient for motor function, 1 patient for respiratory function, 2 patients for swallowing or feeding ability for age), AEs (2 patients), parent's/caregiver's/patient's decision (2 patients), availability of alternative treatment (1 patient), and another reason (1 patient). None of the patients showed loss of developmental milestones after OA infusion.

[c] Four patients were ≥24 months old. Although the data includes results for patients aged ≥24 months at the time of OA infusion due to removal of private information (month and date of birth) used to calculate age, it was confirmed that OA was administered at <24 months of age in all patients.

TABLE S5. Characteristics of patients diagnosed with SMA following newborn screening.

Patient [a]	Therapy	Number of copies of the *SMN2* gene	SMA symptoms at diagnosis	SMA symptoms	Age at SMA diagnosis, months	SMA symptoms at enrollment	Interval between genetic confirmation of SMA and start of initial therapy (R, N, or OA), days	Interval between genetic confirmation of SMA and start of OA infusion, days
A	Bridge to OA infusion	2	Yes	Hypotonia, pneumonia or respiratory symptoms	1	Yes	2 (N)	115
B	Bridge to OA infusion	2	No	–	1	No	3 (N)	228
C	Bridge to OA infusion	3	No	–	3	No	2 (N)	10
D	Combination with OA infusion	2	Yes	Hypotonia, limb weakness	0	Yes	1 (N)	10
E	Combination with OA infusion	3	Yes	Hypotonia, limb weakness, tongue fasciculations, swallowing or feeding difficulties	2	Yes	0 (R)	22
F	OA infusion monotherapy	3	No	–	1	No	15 (OA)	15
G	OA infusion monotherapy	2	No	–	1	Yes	16 (OA)	16
H	OA infusion monotherapy	3	No	–	1	No	13 (OA)	13
I	OA infusion monotherapy	3	No	–	0	No	23 (OA)	23
J	Switch to OA infusion	3	No	–	0	No	9 (N)	246

Abbreviations: N, nusinersen; OA, onasemnogene abeparvovec; R, risdiplam; SMA, spinal muscular atrophy; *SMN2,* survival motor neuron 2.

[a] Patient identifiers removed to preserve anonymity (letters do not necessarily correspond to those used in **Table S8**).

TABLE S6. TEAEs according to the age at OA infusion, by System Organ Class and Preferred Term (in ≥5% of patients).

System Organ Class<br>Preferred Term	Age at OA infusion<br><3 months<br>(*N* = 14)	≥3 to <6 months<br>(*N* = 9)	≥6 to <12 months<br>(*N* = 22)	≥12 to <24 months<br>(*N* = 31)	≥24 months<br>(*N* = 4)	All patients<br>(*N* = 80)
Any TEAE	14 (100.0)	9 (100.0)	22 (100.0)	31 (100.0)	4 (100.0)	80 (100.0)
Blood and lymphatic system disorders	0	1 (11.1)	2 (9.1)	9 (29.0)	1 (25.0)	13 (16.3)
Thrombocytopenia	0	1 (11.1)	2 (9.1)	5 (16.1)	1 (25.0)	9 (11.3)
Thrombotic microangiopathy	0	0	0	4 (12.9)	0	4 (5.0)
Congenital, familial and genetic disorders	2 (14.3)	0	3 (13.6)	0	0	5 (6.3)
Cryptorchism	2 (14.3)	0	2 (9.1)	0	0	4 (5.0)
Gastrointestinal disorders	4 (28.6)	6 (66.7)	14 (63.6)	13 (41.9)	3 (75.0)	40 (50.0)
Vomiting	4 (28.6)	5 (55.6)	10 (45.5)	11 (35.5)	2 (50.0)	32 (40.0)
Gastric fistula	1 (7.1)	2 (22.2)	3 (13.6)	0	0	6 (7.5)
Nausea	0	0	1 (4.5)	3 (9.7)	1 (25.0)	5 (6.3)
General disorders and administration site conditions	9 (64.3)	7 (77.8)	20 (90.9)	25 (80.6)	4 (100.0)	65 (81.3)
Pyrexia	9 (64.3)	7 (77.8)	20 (90.9)	25 (80.6)	4 (100.0)	65 (81.3)
Hepatobiliary disorders	3 (21.4)	2 (22.2)	4 (18.2)	2 (6.5)	1 (25.0)	12 (15.0)
Hepatic function abnormal	3 (21.4)	2 (22.2)	4 (18.2)	2 (6.5)	1 (25.0)	12 (15.0)
Infections and infestations	5 (35.7)	8 (88.9)	11 (50.0)	19 (61.3)	1 (25.0)	44 (55.0)
Pneumonia	2 (14.3)	4 (44.4)	1 (4.5)	6 (19.4)	0	13 (16.3)
Upper respiratory tract infection	3 (21.4)	2 (22.2)	3 (13.6)	5 (16.1)	0	13 (16.3)
Pneumonia aspiration	2 (14.3)	2 (22.2)	2 (9.1)	6 (19.4)	0	12 (15.0)
Bronchitis	0	1 (11.1)	4 (18.2)	3 (9.7)	0	8 (10.0)
COVID-19	2 (14.3)	2 (22.2)	3 (13.6)	1 (3.2)	0	8 (10.0)
Pneumonia respiratory syncytial viral	1 (7.1)	1 (11.1)	1 (4.5)	3 (9.7)	0	6 (7.5)
Respiratory syncytial virus infection	1 (7.1)	1 (11.1)	0	3 (9.7)	0	5 (6.3)
Investigations	12 (85.7)	8 (88.9)	20 (90.9)	28 (90.3)	4 (100.0)	72 (90.0)
Aspartate aminotransferase increased	7 (50.0)	5 (55.6)	13 (59.1)	25 (80.6)	3 (75.0)	53 (66.3)
Alanine aminotransferase increased	6 (42.9)	5 (55.6)	13 (59.1)	25 (80.6)	3 (75.0)	52 (65.0)
Platelet count decreased	3 (21.4)	5 (55.6)	10 (45.5)	19 (61.3)	3 (75.0)	40 (50.0)
Blood lactate dehydrogenase increased	0	4 (44.4)	7 (31.8)	9 (29.0)	1 (25.0)	21 (26.3)
Serum ferritin increased	1 (7.1)	3 (33.3)	5 (22.7)	4 (12.9)	2 (50.0)	15 (18.8)
Troponin I increased	6 (42.9)	3 (33.3)	2 (9.1)	2 (6.5)	1 (25.0)	14 (17.5)
Blood creatine phosphokinase increased	1 (7.1)	1 (11.1)	1 (4.5)	4 (12.9)	1 (25.0)	8 (10.0)

Hepatic enzyme increased	1 (7.1)	2 (22.2)	2 (9.1)	3 (9.7)	0	8 (10.0)
White blood cell count decreased	2 (14.3)	2 (22.2)	3 (13.6)	0	0	7 (8.8)
Weight decreased	0	2 (22.2)	0	4 (12.9)	0	6 (7.5)
Metabolism and nutrition disorders	2 (14.3)	3 (33.3)	6 (27.3)	9 (29.0)	1 (25.0)	21 (26.3)
Decreased appetite	0	1 (11.1)	5 (22.7)	7 (22.6)	1 (25.0)	14 (17.5)
Musculoskeletal and connective tissue disorders	0	3 (33.3)	5 (22.7)	4 (12.9)	0	12 (15.0)
Scoliosis	0	3 (33.3)	4 (18.2)	3 (9.7)	0	10 (12.5)
Respiratory, thoracic and mediastinal disorders	5 (35.7)	2 (22.2)	1 (4.5)	5 (16.1)	0	13 (16.3)
Acute respiratory failure	1 (7.1)	1 (11.1)	0	2 (6.5)	0	4 (5.0)
Atelectasis	0	1 (11.1)	0	3 (9.7)	0	4 (5.0)
Vascular disorders	1 (7.1)	1 (11.1)	0	4 (12.9)	0	6 (7.5)
Kawasaki's disease	1 (7.1)	1 (11.1)	0	2 (6.5)	0	4 (5.0)

Note: Values are *n* (%) of patients.

Abbreviations: OA, onasemnogene abeparvovec; TEAE, treatment-emergent adverse event.

TABLE S7. TEAEs according to the timing of the first event after OA infusion, by System Organ Class and Preferred Term (in ≥5% of patients).

System Organ Class<br>Preferred Term	Timing of onset of first TEAEs after OA infusion							All patients
	≤2 weeks<br>(*N* = 80)	>2 to ≤ 4 weeks<br>(*N* = 80)	>4 weeks to ≤3 months<br>(*N* = 80)	>3 to ≤6 months<br>(*N* = 80)	>6 to ≤12 months<br>(*N* = 79)	>12 to ≤24 months<br>(*N* = 78)	> 24 months<br>(*N* = 70)	(*N* = 80)
Any TEAE	77 (96.3)	19 (23.8)	22 (27.5)	19 (23.8)	26 (32.9)	22 (28.2)	19 (27.1)	80 (100.0)
Blood and lymphatic system disorders	13 (16.3)	0	0	0	0	1 (1.3)	1 (1.4)	13 (16.3)
Thrombocytopenia	9 (11.3)	0	0	0	0	0	0	9 (11.3)
Thrombotic microangiopathy	4 (5.0)	0	0	0	0	0	0	4 (5.0)
Congenital, familial and genetic disorders	0	0	0	0	3 (3.8)	1 (1.3)	1 (1.4)	5 (6.3)
Cryptorchism	0	0	0	0	3 (3.8)	0	1 (1.4)	4 (5.0)
Gastrointestinal disorders	33 (41.3)	2 (2.5)	2 (2.5)	1 (1.3)	1 (1.3)	3 (3.8)	5 (7.1)	40 (50.0)
Gastric fistula	0	0	0	1 (1.3)	0	2 (2.6)	3 (4.3)	6 (7.5)
Nausea	5 (6.3)	0	0	0	0	0	0	5 (6.3)
Vomiting	30 (37.5)	1 (1.3)	0	0	0	0	1 (1.4)	32 (40.0)
General disorders and administration site conditions	64 (80.0)	1 (1.3)	2 (2.5)	1 (1.3)	0	0	0	65 (81.3)
Pyrexia	64 (80.0)	0	1 (1.3)	0	0	0	0	65 (81.3)
Hepatobiliary disorders	9 (11.3)	0	3 (3.8)	0	0	1 (1.3)	0	12 (15.0)
Hepatic function abnormal	9 (11.3)	0	2 (2.5)	0	0	1 (1.3)	0	12 (15.0)
Infections and infestations	3 (3.8)	3 (3.8)	10 (12.5)	12 (15.0)	17 (21.5)	15 (19.2)	14 (20.0)	44 (55.0)
Bronchitis	0	1 (1.3)	0	2 (2.5)	1 (1.3)	4 (5.1)	0	8 (10.0)
COVID-19	0	0	1 (1.3)	0	3 (3.8)	1 (1.3)	3 (4.3)	8 (10.0)
Pneumonia	0	0	0	0	2 (2.5)	5 (6.4)	6 (8.6)	13 (16.3)
Pneumonia aspiration	2 (2.5)	1 (1.3)	0	3 (3.8)	0	4 (5.1)	2 (2.9)	12 (15.0)
Pneumonia respiratory syncytial viral	0	0	2 (2.5)	2 (2.5)	2 (2.5)	0	0	6 (7.5)
Respiratory syncytial virus infection	0	0	1 (1.3)	0	2 (2.5)	2 (2.6)	0	5 (6.3)
Upper respiratory tract infection	0	0	2 (2.5)	3 (3.8)	5 (6.3)	1 (1.3)	2 (2.9)	13 (16.3)

Investigations	72 (90.0)	10 (12.5)	6 (7.5)	2 (2.5)	2 (2.5)	0	0	72 (90.0)
Alanine aminotransferase increased	46 (57.5)	2 (2.5)	4 (5.0)	0	0	0	0	52 (65.0)
Aspartate aminotransferase increased	51 (63.8)	1 (1.3)	1 (1.3)	0	0	0	0	53 (66.3)
Blood creatine phosphokinase increased	6 (7.5)	0	1 (1.3)	1 (1.3)	0	0	0	8 (10.0)
Blood lactate dehydrogenase increased	21 (26.3)	0	0	0	0	0	0	21 (26.3)
Hepatic enzyme increased	7 (8.8)	0	0	1 (1.3)	0	0	0	8 (10.0)
Platelet count decreased	40 (50.0)	0	0	0	0	0	0	40 (50.0)
Serum ferritin increased	15 (18.8)	0	0	0	0	0	0	15 (18.8)
Troponin I increased	12 (15.0)	2 (2.5)	0	0	0	0	0	14 (17.5)
Weight decreased	3 (3.8)	1 (1.3)	0	0	2 (2.5)	0	0	6 (7.5)
White blood cell count decreased	7 (8.8)	0	0	0	0	0	0	7 (8.8)
Metabolism and nutrition disorders	18 (22.5)	0	1 (1.3)	1 (1.3)	1 (1.3)	1 (1.3)	0	21 (26.3)
Decreased appetite	14 (17.5)	0	0	0	0	0	0	14 (17.5)
Musculoskeletal and connective tissue disorders	0	0	1 (1.3)	4 (5.0)	6 (7.6)	1 (1.3)	1 (1.4)	12 (15.0)
Scoliosis	0	0	1 (1.3)	2 (2.5)	5 (6.3)	1 (1.3)	1 (1.4)	10 (12.5)
Respiratory, thoracic and mediastinal disorders	0	2 (2.5)	3 (3.8)	2 (2.5)	2 (2.5)	4 (5.1)	5 (7.1)	13 (16.3)
Acute respiratory failure	0	0	0	0	1 (1.3)	3 (3.8)	0	4 (5.0)
Atelectasis	0	0	0	2 (2.5)	0	1 (1.3)	1 (1.4)	4 (5.0)
Vascular disorders	2 (2.5)	0	0	0	1 (1.3)	0	3 (4.3)	6 (7.5)
Kawasaki's disease	0	0	0	0	1 (1.3)	0	3 (4.3)	4 (5.0)

Note: Values are *n* (%) of patients.

Abbreviations: OA, onasemnogene abeparvovec; TEAE, treatment-emergent adverse event.

TABLE S8. Characteristics of patients with tracheostomy (before or after OA infusion) [a].

Patient [b]	Therapy	Number of copies of the *SMN2* gene	Age at OA infusion (months)	Time from initial therapy to tracheostomy (months)	Age (months) at:		Reason for tracheostomy
					Tracheostomy	Data cutoff	
A	Bridge to OA infusion	2	7	1.1	2.9	66.3	Disease progression without acute cause
B	Bridge to OA infusion	2	7	2.1	5.1	47.5	Pneumonia
C	Bridge to OA infusion	2	7	58.0	58.7	63.6	Aspiration pneumonia
D	Combination with OA infusion	2	14	59.2	65.7	73.3	Disease progression without acute cause
E	Combination with OA infusion	3	3	17.3	19.8	32.7	Respiratory failure associated with RSV infection
F	Combination with OA infusion	2	4	7.8	10.5	41.2	Pneumonia
G	OA monotherapy	2	2	17.2	19.5	44.7	Upper respiratory illness
H	Switch to OA therapy	2	23	3.4	8.8	80.4	Disease progression without acute cause
I	Switch to OA therapy	2	24	6.5	8.1	82.7	Upper respiratory illness
J	Switch to OA therapy	2	7	14.7	16.6	22.8	Disease progression without acute cause
K	Transient add-on	2	2	23.1	24.9	60.3	Pneumonia

Abbreviations: OA, onasemnogene abeparvovec; PMS, post-marketing surveillance; RSV, respiratory syncytial virus; *SMN2*, survival motor neuron 2.

[a] All patients were continuing the PMS at the cutoff date.

[b] Patient identifiers removed to preserve anonymity (letters do not necessarily correspond to those used in **Table S5**).

TABLE S9. Proportions of patients with changes in HFMSE and HINE-2 scores according to newborn screening status.

	Patients screened for SMA as a newborn (*N* = 10)	Patients with clinical diagnosis (*N* = 70)	All patients (*N* = 80)
HFMSE			
Evaluable patients, *n* [a]	1	29	30
Score increased by ≥3 points, *n* (%)	0	25 (86.2)	25 (83.3)
Score maintained within 3 points in either direction, *n* (%)	1 (100)	7 (24.1)	8 (26.7)
Score decreased by ≥3 points, *n* (%)	0	1 (3.4)	1 (3.3)
HINE-2			
Evaluable patients, *n* [a]	2	18	20
Score increased by ≥2 points, *n* (%)	2 (100)	16 (88.9)	18 (90.0)
Score increased by ≥1 point, *n* (%)	2 (100)	16 (88.9)	18 (90.0)

Abbreviations: HFMSE, Hammersmith Functional Motor Scale Expanded; HINE-2, Hammersmith Infant Neurological Examination Section 2; SMA, spinal muscular atrophy.

[a] Among patients with at least two assessments, with the second assessment after onasemnogene abeparvovec treatment.

TABLE S10. Proportions of patients with changes in HFMSE and HINE-2 scores according to the presence of symptoms at diagnosis.

	**Asymptomatic (*N* = 10)**	**Symptomatic (*N* = 70)**	**All patients (*N* = 80)**
HFMSE			
Evaluable patients, *n* [a]	4	26	30
Score increased by ≥3 points, *n* (%)	2 (50.0)	23 (88.5)	25 (83.3)
Score maintained within 3 points in either direction, *n* (%)	2 (50.0)	6 (23.1)	8 (26.7)
Score decreased by ≥3 points, *n* (%)	0	1 (3.8)	1 (3.3)
HINE-2			
Evaluable patients, *n* [a]	3	17	20
Score increased by ≥2 points, *n* (%)	3 (100)	15 (88.2)	18 (90.0)
Score increased by ≥1 point, *n* (%)	3 (100)	15 (88.2)	18 (90.0)

Abbreviations: HFMSE, Hammersmith Functional Motor Scale Expanded; HINE-2, Hammersmith Infant Neurological Examination Section 2.

[a] Among patients with at least two assessments, with the second assessment after onasemnogene abeparvovec treatment.

TABLE S11. Proportions of patients with changes in HFMSE and HINE-2 scores according to the number of copies of the *SMN2* gene.

	**2 copies (*N* = 40)**	**3 copies (*N* = 40)**	**All patients (*N* = 80)**
HFMSE			
Evaluable patients, *n* [a]	12	18	30
Score increased by ≥3 points, *n* (%)	11 (91.7)	14 (77.8)	25 (83.3)
Score maintained within 3 points in either direction, *n* (%)	4 (33.3)	4 (22.2)	8 (26.7)
Score decreased by ≥3 points, *n* (%)	0	1 (5.6)	1 (3.3)
HINE-2			
Evaluable patients, *n* [a]	11	9	20
Score increased by ≥2 points, *n* (%)	10 (90.9)	8 (88.9)	18 (90.0)
Score increased by ≥1 point, *n* (%)	10 (90.9)	8 (88.9)	18 (90.0)

Abbreviations: HFMSE, Hammersmith Functional Motor Scale Expanded; HINE-2, Hammersmith Infant Neurological Examination Section 2; *SMN2*, survival motor neuron 2.

[a] Among patients with at least two assessments, with the second assessment after onasemnogene abeparvovec treatment.
